# Depletion of B2 but Not B1a B Cells in BAFF Receptor-Deficient ApoE^−/−^ Mice Attenuates Atherosclerosis by Potently Ameliorating Arterial Inflammation

**DOI:** 10.1371/journal.pone.0029371

**Published:** 2012-01-04

**Authors:** Tin Kyaw, Christopher Tay, Hamid Hosseini, Peter Kanellakis, Tahlia Gadowski, Fabeinne MacKay, Peter Tipping, Alex Bobik, Ban-Hock Toh

**Affiliations:** 1 Vascular Biology and Atherosclerosis Laboratory, Baker IDI Heart and Diabetes Institute, Victoria, Australia; 2 Centre for Inflammatory Diseases, Department of Medicine, Southern Clinical School, Faculty of Medicine, Nursing and Health Sciences, Monash University, Victoria, Australia; 3 Department of Immunology, Central Clinical School, Faculty of Medicine, Nursing and Health Sciences, Monash University, Victoria, Australia; Maastricht University, The Netherlands

## Abstract

We have recently identified conventional B2 cells as atherogenic and B1a cells as atheroprotective in hypercholesterolemic ApoE^−/−^ mice. Here, we examined the development of atherosclerosis in BAFF-R deficient ApoE^−/−^ mice because B2 cells but not B1a cells are selectively depleted in BAFF-R deficient mice. We fed BAFF-R^−/−^ ApoE^−/−^ (*BaffR.ApoE* DKO) and BAFF-R^+/+^ApoE^−/−^ (*ApoE* KO) mice a high fat diet (HFD) for 8-weeks. B2 cells were significantly reduced by 82%, 81%, 94%, 72% in blood, peritoneal fluid, spleen and peripheral lymph nodes respectively; while B1a cells and non-B lymphocytes were unaffected. Aortic atherosclerotic lesions assessed by oil red-O stained-lipid accumulation and CD68+ macrophage accumulation were decreased by 44% and 50% respectively. B cells were absent in atherosclerotic lesions of *BaffR.ApoE* DKO mice as were IgG1 and IgG2a immunoglobulins produced by B2 cells, despite low but measurable numbers of B2 cells and IgG1 and IgG2a immunoglobulin concentrations in plasma. Plasma IgM and IgM deposits in atherosclerotic lesions were also reduced. BAFF-R deficiency in ApoE^−/−^ mice was also associated with a reduced expression of VCAM-1 and fewer macrophages, dendritic cells, CD4+ and CD8+ T cell infiltrates and PCNA+ cells in lesions. The expression of proinflammatory cytokines, TNF-α, IL1-β and proinflammatory chemokine MCP-1 was also reduced. Body weight and plasma cholesterols were unaffected in *BaffR.ApoE* DKO mice. Our data indicate that B2 cells are important contributors to the development of atherosclerosis and that targeting the BAFF-R to specifically reduce atherogenic B2 cell numbers while preserving atheroprotective B1a cell numbers may be a potential therapeutic strategy to reduce atherosclerosis by potently reducing arterial inflammation.

## Introduction

Atherosclerosis is a chronic inflammatory disease of large arteries initiated by lipid entry. Despite the therapeutic application of lipid-lowering statins; atherosclerosis-related vascular disease remains the major cause of mortality from heart attacks and strokes. New therapies to attenuate the chronic inflammation in atherosclerosis are therefore urgently sought that can be combined with current lipid-control medications and healthy life-style adaptation [1], [2]. B cells together with other immune cells are implicated in the pathogenesis and progression of atherosclerosis. Previous studies have suggested that these B cells are atheroprotective [3], [4]. However, in a major paradigm shift, we and Ait-Oufella et al have reported that these B cells can be pathogenic because their depletion by anti-CD20 monoclonal antibody ameliorated atherosclerosis in ApoE^−/−^ and LDLR^−/−^ mice [5], [6]. In adoptive transfer experiments we have identified conventional B2 B cells as an atherogenic B cell subset and peritoneal B1a B cells as an atheroprotective B cell subset in atherosclerosis [5], [7]. Consequentially, we have proposed a potential therapeutic strategy for atherosclerosis based on selective depletion of atherogenic B2 B cells without depleting atheroprotective peritoneal B1a B cells [8].

B-cell activating factor (BAFF), also known as BlyS, TALL-1, zTNF4 and THANK, is a member of the TNF superfamily (TNFSF13B) that is produced by myeloid cells, non-lymphoid cells and epithelial cells [9]. BAFF is required for maturation and survival of B2 cells [10]. Its biological activities are mediated by three receptors, BAFF-receptor (BAFF-R; TNFRSF13C), transmembrane activator-calcium modulator and cyclophilin ligand interactor (TACI; TNFRSF13B) and B-cell maturation antigen (BCMA; TNFRSF17) [11]. While TACI and BCMA can also interact with the BAFF homologue, a proliferation-inducing ligand (APRIL; TNFSF13), BAFF-R is expressed by all mature B cells and only binds BAFF to initiate signaling that is crucial for B cell development and survival [12].

Mice with genetically disrupted BAFF-R gene and spontaneous mutation in the BAFF-R gene show a significant reduction in mature B2 cells without affecting B1a B cells [13], [14]. Therefore BAFF-R has properties that are suitable for therapeutic targeting in atherosclerosis. Here, we have examined the role of BAFF-R in atherosclerosis using ApoE^−/−^ mice deficient in BAFF-R. We report that atherosclerosis and arterial inflammation is markedly reduced in hypercholesterolemic BAFF-R deficient ApoE^−/−^ mice.

## Results

### Generation and characteristics of BAFF-R-deficient ApoE^−/−^ mouse

**Figure 1:**
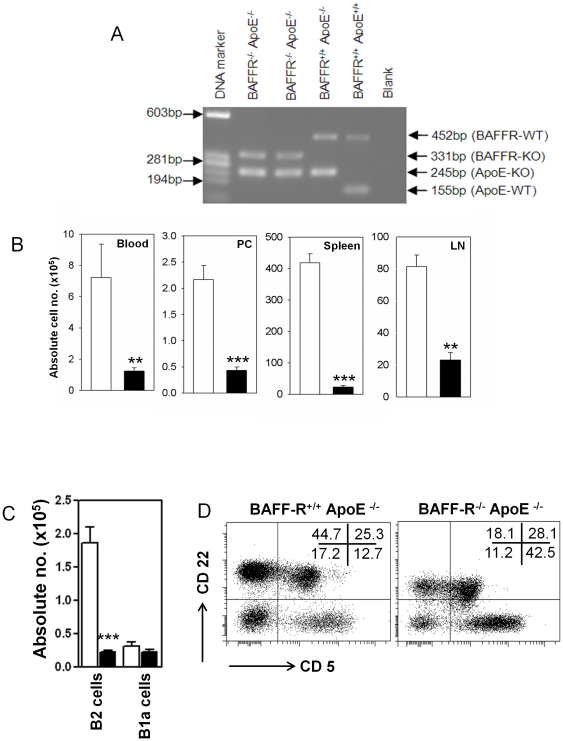
BAFF-R deficiency attenuates selectively conventional B2 cells, not peritoneal B1a cells. (**A**) PCR analysis of BAFF-R gene disruption was performed using DNA extracted from tails of BAFF-R^−/−^ ApoE^−/−^, BAFF-R^+/+^ ApoE^−/−^ and BAFF-R^+/+^ ApoE^+/+^ mice. (**B**) *BaffR.ApoE* DKO mice showed that CD22^+^ B cells were lowered in peripheral blood, peritoneal cavity, spleen and peripheral lymph nodes. (**C**) FACS analysis on peritoneal cavity revealed that depleted B cells were conventional CD22+ CD5- B2 cells, not CD22+ CD5+ B1a cells comparing to *ApoE* KO mice. (**D**) A representative FACS analysis of peritoneal cavity showed that only conventional CD22+ CD5- B2 cell population (left upper quadrant) was decreased in *BaffR.ApoE* DKO and peritoneal CD22+ CD5+ B1a cell (right upper quadrant) was unaffected. Open bar = *ApoE* KO; Black bar = *BaffR.ApoE* DKO; n = 9–11 mice; **: *p*<0.01, ***: *p*<0.001.

We generated BAFF-R^−/−^ ApoE^−/−^ (*BaffR.ApoE* DKO) mice by crossing C57Bl/6 BAFF-R^−/−^ mice with atherosclerosis-prone C57Bl/6 ApoE^−/−^ (*ApoE* KO) mice. Genotypes of *BaffR.ApoE* DKO and *ApoE* KO mice were verified by PCR (**Figure 1 A**). *BaffR.ApoE* DKO and *ApoE* KO mice were fed a high fat diet (HFD) containing 21% fat and 0.15% cholesterol (Specialty Feed, Western Australia) for eight weeks to study the role of BAFF-R in atherosclerosis.

Genes encoding BAFF-R disrupted by spontaneous mutation [13] or gene-targeted depletion [14] showed a decrease in mature B cells. To confirm similar effects in the *BaffR.ApoE* DKO mice, CD22+ B cell population was analyzed in different tissues at the end of 8-week HFD period. We found that B cells were significantly reduced in peripheral blood, peritoneal cavity, spleen and lymph node by 82% (*p*<0.01), 81% (*p*<0.001), 94% (*p*<0.001) and 72% (*p*<0.01) respectively in BAFF-R-deficient ApoE^−/−^ mice (**Figure 1 B**).

Although BAFF-R is necessary for maturation of B cells from transitional B cells to follicular B cells and Marginal Zone B cells, BAFF-R deletion has either no or minimal effects on the B1 B cell linage [13], [14]. In accordance with the literature, peritoneal CD22+ CD5+ B1a cell population was not affected by depletion of BAFF-R gene in ApoE^−/−^ mice (*p*>0.05; **Figure 1 C and D**). FACS analysis of splenic cells showed that non-B cell populations, including CD4+CD25+ Foxp3 regulatory T cells were unaffected in BAFF-R-deficient ApoE^−/−^ mice (*p*>0.05; **Table 1**), consistent with a previous report [14].

**Table 1:** Non-B lymphocyte populations in spleen (×10^6^).

	Non-B cells	*ApoE* KO	*BaffR.ApoE* DKO
Spleen	CD4+ T cells	17.13±2.86	18.97±3.15
	CD8+ T cells	13.91±2.33	19.42±3.29
	NK cells	1.44±0.09	1.02±0.11
	NKT cells	1.00±0.04	0.8±0.13
	Regulatory T cells	2.67±0.42	2.31±0.04

At the end of 8 week HFD, spleen cells are analyzed for non-B lymphocyte populations (n = 9–11 mice).

Hypercholesterolemia is an initiating factor in the pathogenesis of atherosclerosis. Plasma lipid analysis showed that there was no difference in plasma total cholesterol, HDL-cholesterol, VLDL/LDL-cholesterol and triglycerides between BAFF-R-deficient and –competent ApoE^−/−^ mice fed a HFD for eight weeks (*p*>0.05; **Table 2**). Similarly, BAFF-R depletion did not affect the body weight in ApoE^−/−^ mice (*p*>0.05; **Table 2**).

**Table 2:** Factors contributing in atherosclerosis pathogenesis.

	*ApoE* KO	*BaffR.ApoE* DKO
Bodyweight	30.92±0.59	28.97±1.06
Total cholesterol	19.85±2.03	18.56±1.74
HDL cholesterol	3.54±0.33	2.95±0.27
VLDL/LDL cholesterol	15.35±1.77	13.42±1.25
Triglycerides	2.67±0.58	4.21±1.00

At the end of 8 week HFD, body weight and plasma lipids were analyzed (see text) (n = 9–11 mice).

### *BaffR.ApoE* DKO mice exhibit reduced atherosclerosis

**Figure 2:**
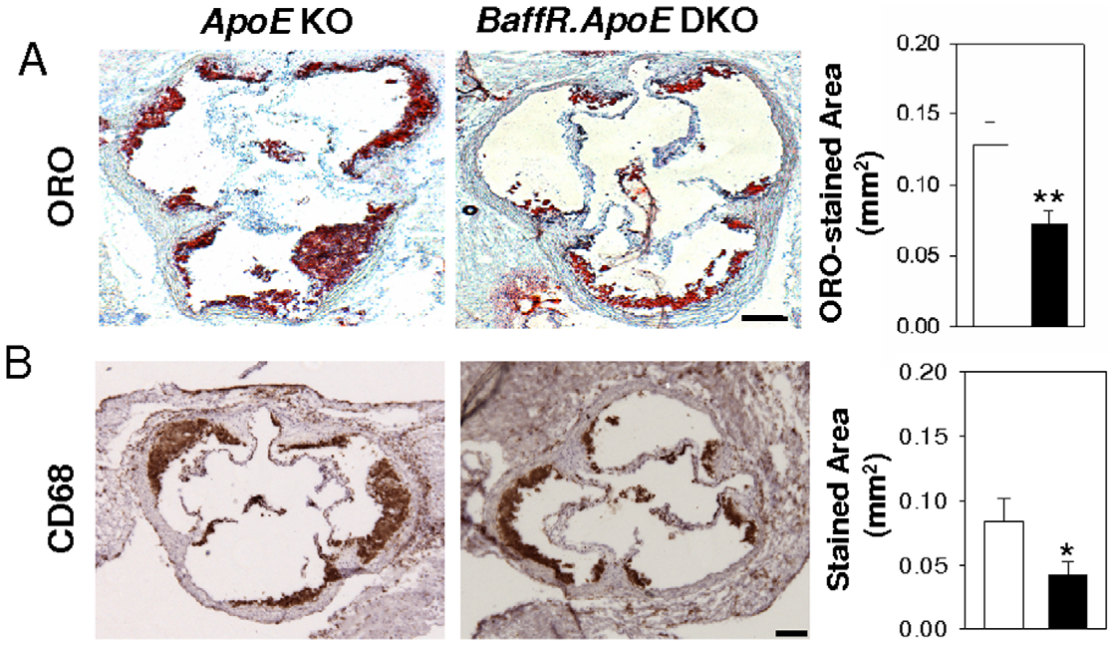
Deficiency of BAFF-R attenuates atherosclerosis. After feeding a HFD for eight weeks, *BaffR.ApoE* DKO showed decreased atherosclerosis at aortic sinus as assessed by (**A**) oil-red O stained lipid accumulation and (**B**) CD68+ macrophage accumulation compared to *ApoE* KO. Open bar = *ApoE* KO; Black bar = *BaffR.ApoE* DKO; n = 9–11 mice; scale bar = 100 µm; *: *p*<0.05, **: *p*<0.01.

Next, we asked if the deficiency in BAFF-R influences the development of atherosclerosis in hypercholesterolemic ApoE^−/−^ mice. Assessment of atherosclerotic lesions after feeding mice a HFD by oil red-O stained lipid accumulation showed that *BaffR.ApoE* DKO mice had a significant ∼44% reduction in aortic atherosclerosis compared to *ApoE* KO mice (*p*<0.01; **Figure 2 A**). We found that a similar ∼50% reduction in atherosclerosis as assessed by CD68-stained macrophage accumulation in *BaffR.ApoE* DKO mice (*p*<0.05; **Figure 2 B**).

### Lack of B cells and immunoglobulins G in atherosclerotic lesions of *BaffR.ApoE* DKO mice

**Figure 3:**
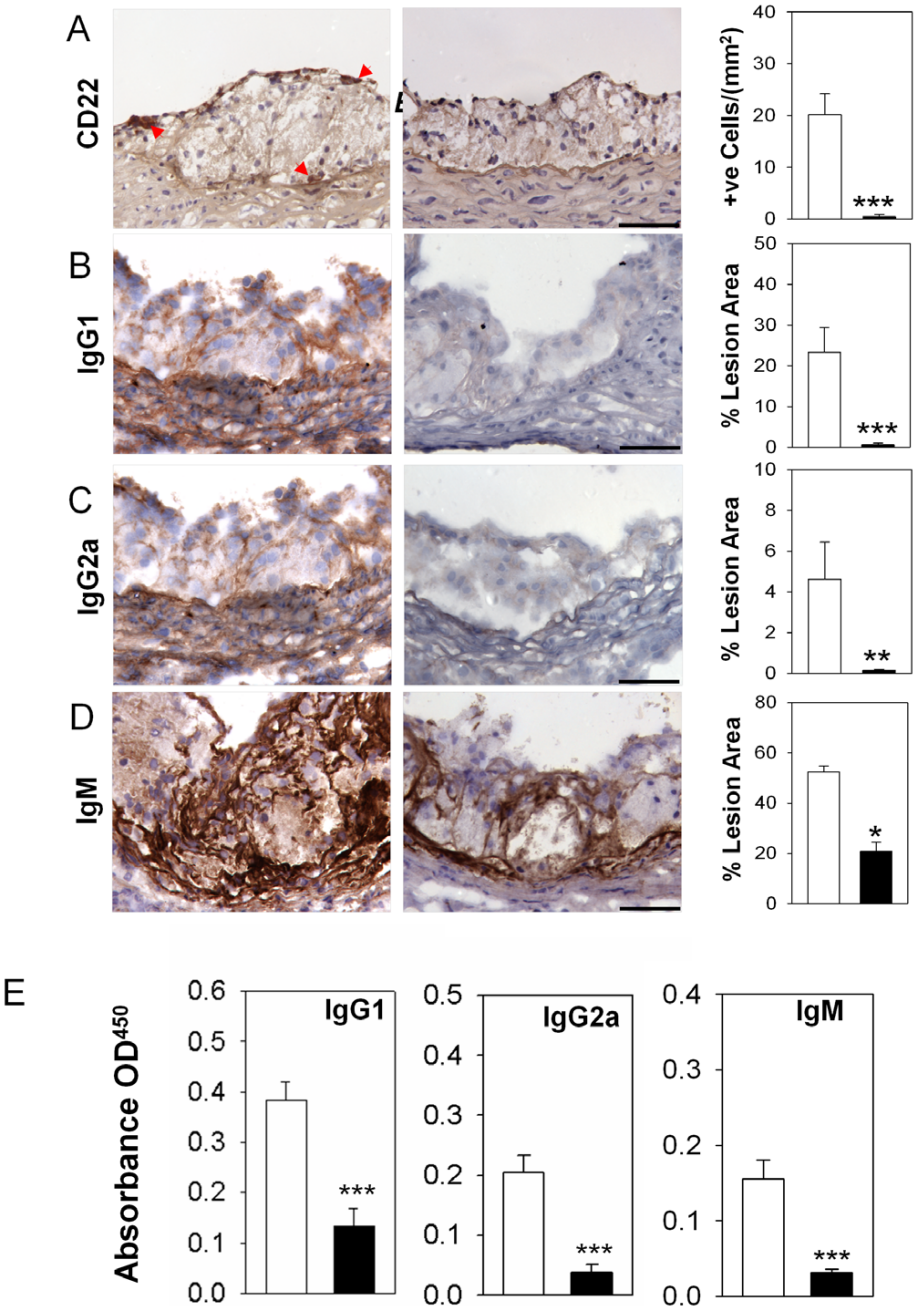
BAFF-R deficiency responsible for loss of B2 cells affects loss of B cells and IgG deposits in atherosclerotic lesion and attenuates plasma IgG levels. (**A**) CD22+ B cells and (**B–D**) immunoglobulins deposits (IgG1, IgG2a and IgM) found in the atherosclerotic lesion of *ApoE* KO mice were not detected in *BaffR.ApoE* DKO mice. (**E**)The plasma IgG1, IgG2a and IgM levels are also significantly decreased in *BaffR.ApoE* DKO but to a lesser extent than in lesions. Open bar = *ApoE* KO; Black bar = *BaffR.ApoE* DKO; n = 9–11 mice; scale bar = 100 µm; **: *p*<0.01, ***: *p*<0.001.

We next determined whether B cell numbers were reduced in lesions of *BaffR.ApoE* DKO mice using anti-CD22 immunohistochemistry. In marked contrast to *ApoE* KO mice, there was no accumulation of B cells in atherosclerotic lesions in *BaffR.ApoE* DKO mice (*p*<0.001; **Figure 3 A**). As IgG1 and IgG2a are produced by B2 cells, we also assessed their accumulation in lesions. Again, in contrast to lesions of *ApoE* KO mice, those of *BaffR.ApoE* DKO mice did not contain detectable quantities of these two immunoglobulins (*p*<0.001 and *p*<0.05 respectively; **Figure 3 B and C**), confirming the marked differences in B cell accumulation between *ApoE* KO and *BaffR.ApoE* DKO mice. To ensure that the reductions in lesion IgG1 and IgG2a were the consequence of markedly different B cell numbers in lesions of the two groups of mice, we also assessed circulating levels of IgG1 and IgG2a. As expected, ELISA results revealed that the plasma levels of IgG1 and IgG2a were decreased by 65% and 82% as the result of BAFF-R gene disruption (both *p*<0.01; **Figure 3 E**).

### BAFF-R deficiency reduced plasma IgM and IgM deposits in lesions

ELISA results revealed that levels of plasma IgM in *BaffR.ApoE* DKO mice were significantly decreased to about ∼80% of plasma IgM levels of ApoE^−/−^ mice (*p*<0.001; **Figure 3 E**). Significant reductions of plasma IgM prompted us to investigate whether IgM deposits were also altered in atherosclerotic lesions of BAFF-R^−/−^ ApoE^−/−^ mice. Indeed, we found that IgM deposits in atherosclerotic lesion were also decreased to 60% in *BaffR.ApoE* DKO mice compared to ApoE^−/−^ mice (*p*<0.01; **Figure 3 D**).

### Decreased T cells and PCNA-positive cells in lesions of *BaffR.ApoE* DKO mice

**Figure 4:**
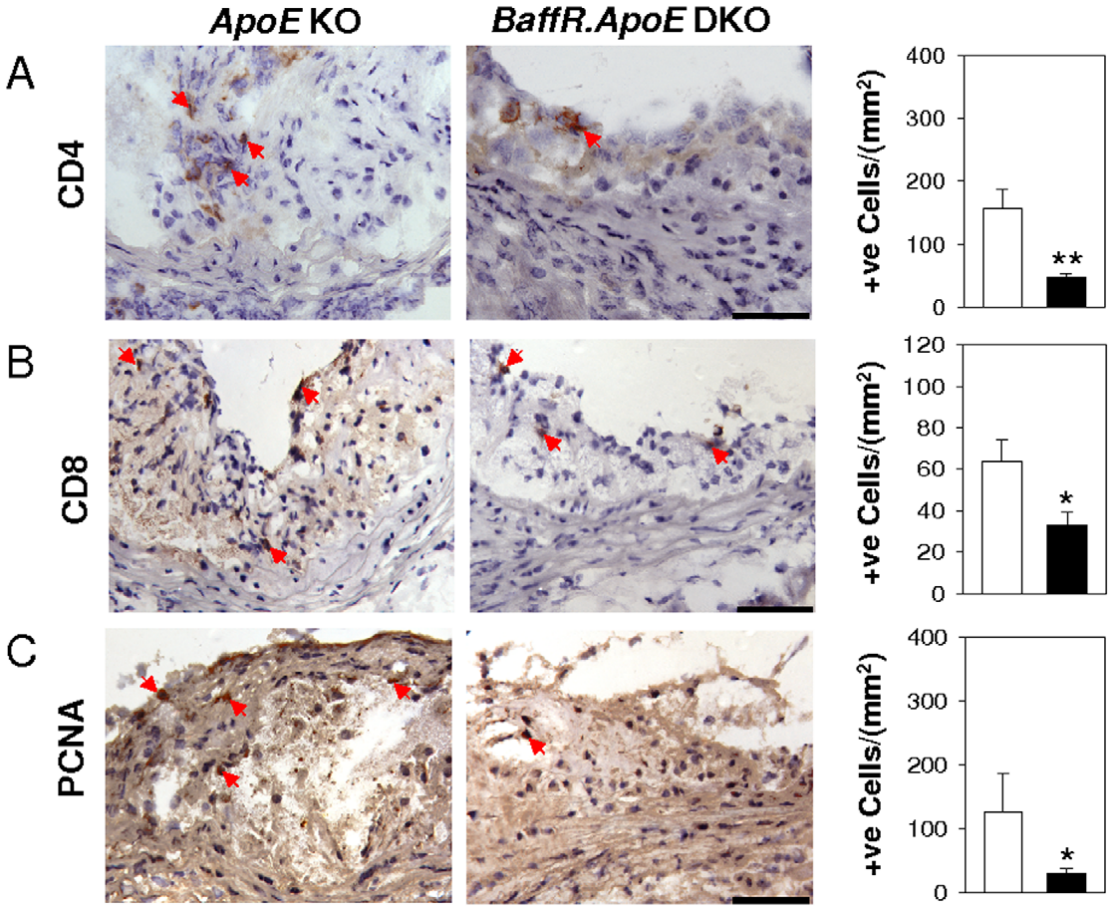
T cell infiltrates and cellular proliferative activity are decreased in atherosclerotic lesions of *BaffR.ApoE* DKO. (**A and B**) T cell infiltrates as assessed by anti-CD4 and anti-CD8 antibodies and (**C**) cellular proliferative activity as assessed by PCNA antibody were reduced by deficiency of BAFF-R. Open bar = *ApoE* KO; Black bar = *BaffR.ApoE* DKO; n = 9–11 mice; scale bar = 100 µm; *: *p*<0.05, **: *p*<0.01.

Next we asked if there were any reductions in T cells and PCNA-positive cells in lesions of *BaffR.ApoE* DKO mice that could account for the reductions in lesion size. CD4+ and CD8+ T cells as well as PCNA+ cells are cellular markers of inflammatory responses and active proliferative activity in atherosclerosis [15]–[17]. Using CD4, CD8 and PCNA-specific antibodies, we found that CD4+ T cells and proliferating PCNA+ cells were reduced by 69% (*p*<0.05) and 76% (*p*<0.05) respectively in lesions of the *BaffR.ApoE* DKO mice compared to *ApoE* KO mice. In addition CD8+ T cell numbers were reduced by 49% (*p*<0.05; **Figure 4**).

### Reductions in VCAM-1 expression and dendritic cells in lesions of BAFF-R^−/−^ ApoE^−/−^ mice

**Figure 5:**
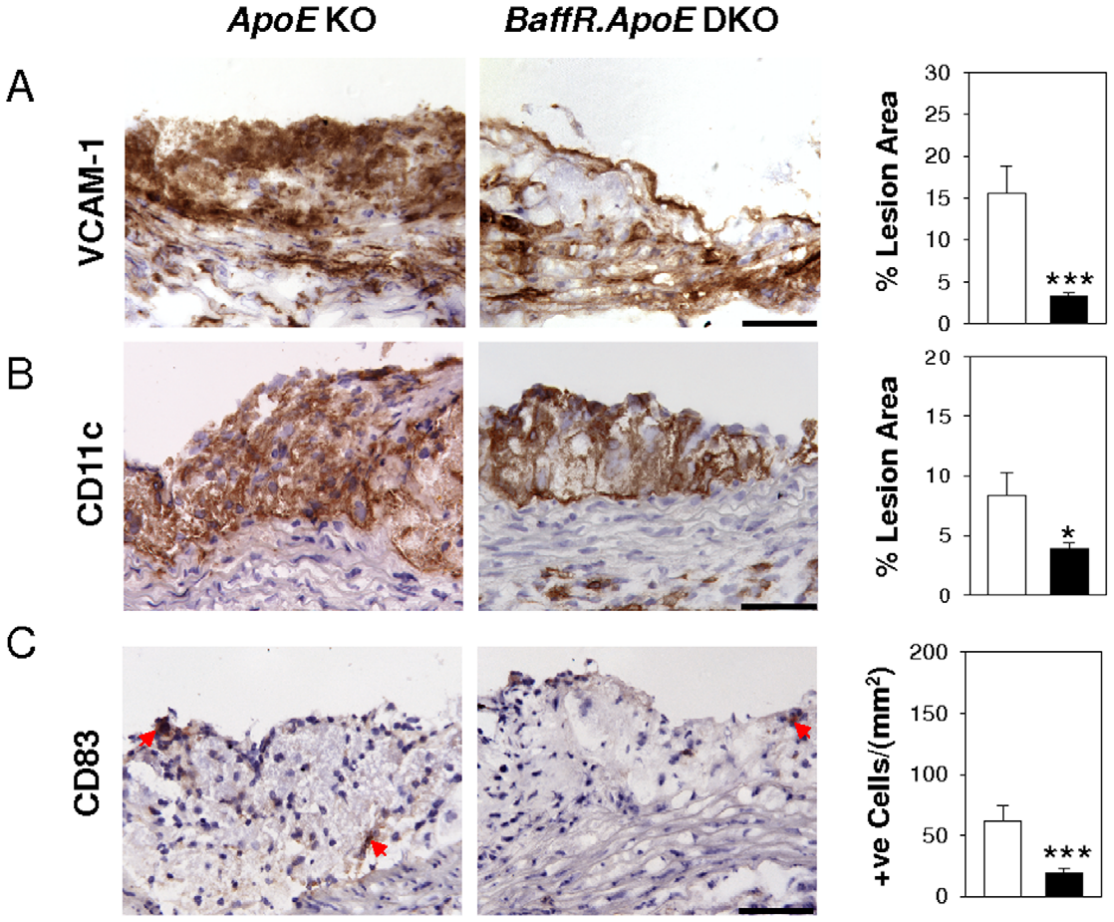
Low expression of adhesion molecule, VCAM-1, is associated with reduction in immature and mature dendritic cells in atherosclerotic lesions of BAFF-R^−/−^ ApoE^−/−^ mice. (**A**) VCAM-1 expression decreased in atherosclerotic lesions by disruption of BAFF-R gene was accompanied by (**B**) decreased immature dendritic cells as assessed by anti-CD11c antibody and (**C**) mature dendritic cells as assessed by anti-CD83 antibody in *BaffR.ApoE* DKO mice. Open bar = *ApoE* KO; Black bar = *BaffR.ApoE* DKO; n = 9–11 mice; scale bar = 100 µm; *: *p*<0.05, ***: *p*<0.001.

We carried out immunohistochemical assays to determine whether expression of VCAM-1 in atherosclerotic lesions was affected by BAFF-R gene disruption. VCAM-1 regulates the migration of leukocytes, including monocyte infiltration into developing lesions [18] and promotes the differentiation of monocytes into macrophages and dendritic cells to sites of inflammation [19]. Data analysis indicated that expression of lesional VCAM-1 in BAFF-R-deficient mice showed a ∼79% decrease compared to BAFF-R-competent mice (*p*<0.001; **Figure 5 A**). We also found that CD11c+ immature dendritic cells and CD83+ mature dendritic cells were reduced by 54% (*p*<0.05) and 68% (*p*<0.001) in *BaffR.ApoE* DKO mice compared to *ApoE* KO mice (**Figure 5 B and C**).

### Decreased inflammatory cytokines and MCP-1 expression in lesions of *BaffR.ApoE* DKO mice

**Figure 6:**
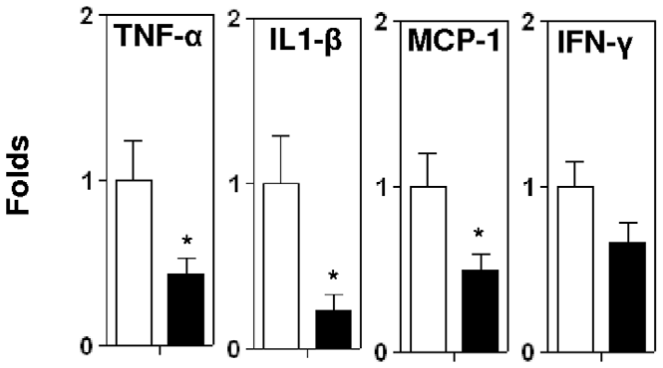
Real-time PCR analysis of proinflammatory cytokines. RNAs extracted from aortic arches were analysed for proinflammatory cytokines, TNF-α, IL1-β, MCP-1 and IFN-γ. *BaffR.ApoE* DKO showed reduced expression of proinflammatory cytokines in aortic arches compared to *ApoE* KO. Open bar = *ApoE* KO; Black bar = *BaffR.ApoE* DKO; n = 9–11 mice; *: *p*<0.05.

IFN-γ expression by CD4+ T cells is reduced by anti-CD20 B cell depletion in atherosclerotic mice [6]. To determine whether IFN-γ and other proinflammatory cytokines and chemokines were also reduced in BAFF-receptor deficient mice, we performed real-time PCR analysis using mRNA extracted from aorta containing atherosclerotic aortic arches. We found that expression of mRNA encoding the proinflammatory cytokines TNF-α and IL1-β as well as monocyte attractant MCP-1 were decreased to 57%, 77% and 51% respectively in *BaffR.ApoE* DKO mice (All *p*<0.05; **Figure 6**). The expression level of IFN-γ was reduced by 24%, but it was not statistically significant (**Figure 6**).

## Discussion

In this study, we have provided evidence that the attenuation of atherosclerosis and decreased inflammatory responses in atherosclerotic lesions in *BaffR.ApoE* DKO mice is linked to a reduced B2 cell population. In accordance with our previous reports [5], [7], the development of atherosclerosis is potently ameliorated in an environment where atherogenic B2 cells but not atheroprotective B1a cells are reduced, as occurs in BAFF-R-deficient ApoE^−/−^ mice.

The vast majority of conventional B2 B cells express BAFF-R which is essential for BAFF mediated maturation as well as their survival. Our data demonstrate that B2 cells are major regulators of atherosclerosis development. These findings are consistent with our previous observation that the adoptive transfer of B2 but not B1a cells to either lymphocyte deficient or B cell deficient mice aggravates atherosclerosis [5]. We suggest that targeting B2 cells via the BAFF receptor that spares B1a cells may be more therapeutically efficacious than B cell depletion strategies using monoclonal antibody to CD20 that depletes both B2 as well as B1a cells [5], [6].

BAFF-R gene defect is associated with a strong, but not complete, reduction of mature B cells, sparing the B1a cell in both spontaneous mutation and knockout mice [13], [14]. B cells contribute to the production of immunoglobulins, particularly subclass IgG type. IgG constitutes about 75% of immunoglobulins in the circulation and is the major Ig class generated in adaptive immune responses. Our finding of reduced circulating immunoglobulin IgG1 and IgG2a in mature B cell-depleted *BaffR.ApoE* DKO mice is consistent with the inability of immature transitional B cells to efficiently differentiate into mature B cells [13], [14].

IgG deposits are reported in both human and animal atherosclerotic lesions [20]–[22]. The origin of IgG is yet to be determined. It could arise from B cells and plasma cells in the intimal or adventitial layer and/or the circulating IgG pool [21], [22]. In our study, both IgG1 and IgG2a detected in both intimal and medial layers in BAFF-R-competent ApoE^−/−^ mice were almost undetectable in BAFF-R-deficient ApoE^−/−^ mice, despite reduced but still easily detectable, levels of these IgGs in the circulation. Concurrently CD22+ B cells in atherosclerotic lesions were reduced to almost none in BAFF-R deficient ApoE^−/−^ mice. Taken together, it is likely that lesion IgG deposits found in BAFF-R-competent ApoE^−/−^ mice are mostly derived from B cell and/or plasma cells located in the intimal or adventitial layers. Plasma IgM levels at the end of the 8 week-HFD were also decreased in *BaffR.ApoE* DKO mice consistent with a previous report [14] and with the suggestion that BAFF-R also has a role in germinal centre responses and in Ig class-switch DNA recombination [14], [23], [24]. The reduction in IgM deposits that we observed in atherosclerotic lesions may be a consequence of the reduced levels of circulating IgM. The residual levels of IgM deposits in atherosclerotic lesions may represent natural IgM produced by peritoneal Bla cells as we have previously reported [7].

Accelerated local inflammation responsible for progression of atherosclerosis and its complications is mediated by atherogenic cells as well as proinflammatory proteins in atherosclerotic lesions [25]. Immune cells such as monocytes, macrophages, dendritic cells and lymphocytes migrate into atherosclerotic lesions, to contribute to inflammation by interacting with other proinflammatory immune cells [16]–[19]. Activated immune cells secrete a range of pro-inflammatory cytokines to intensify the inflammatory process locally. These cytokines promote both their own production and the production of other cytokines and can also target the cells that produce them as well as neighboring cells to promote inflammation [26]. Thus, proinflammatory cytokines produced by macrophages, dendritic cells, CD4+ and CD8+ T cells promote the development and progression of atherosclerosis as positive regulators.

VCAM-1 is an adhesion molecule required for recruitment of lymphocytes and monocytes from the circulation into atherosclerotic lesions [18], [27]–[31]. Monocytes recruited into atherosclerotic lesions further differentiate into macrophages and dendritic cells [32]. Our results suggest that the recruitment and retention of proinflammatory macrophages, dendritic cells and CD4 and CD8 T cells in atherosclerotic lesions are decreased due to low expression of VCAM-1 in *BaffR.ApoE* DKO mice.

Macrophages are major proinflammatory cells in the pathogenesis of atherosclerosis. They appear early in the atherosclerotic lesion to drive the inflammatory process in atherosclerotic lesions. We suggest that the reduced accumulation of macrophages contributes to the attenuated inflammatory responses that result in decreased atherosclerosis. It is also likely that the decreased accumulation of T cells, contributes to the reduced arterial inflammation. CD8+ T cells are important not only in recruitment, differentiation and activation of macrophages in the early stage of inflammatory responses, but also in maintenance of subsequent inflammatory responses [33]. The reduction of CD4 T cells in atherosclerotic lesions may be attributed at least in part to BAFF-R providing co-stimulatory signals [34] that augment Th1 inflammatory responses [35]. CD4+ T cells can activate macrophages to produce pro-inflammatory cytokines such as TNF-α, IL1-β, MCP-1 [36].

Our findings also clearly show that selective deficiency of B2 but not B1a cells is accompanied by reduction in the lesions of, the proinflammatory cytokines TNF-α, IL1-β and MCP-1 [16]–[18], [25]–[31], [33], [36]. We have also previously reported reduced TNF-α in atherosclerotic lesions that accompany the reduced atherosclerosis following B cell depletion by anti-CD20 monoclonal antibody treatment [5]. The reduction in TNF-α appears to be the consequence of depletion of B2 cells as these cells are known to be significant producers of TNF-α [34], [37]. Expression of IFN-γ showed a trend in reduction in *BaffR-ApoE* DKO mice, but the result was not statistically significant. As TNF-α has been reported to upregulate VCAM-1 expression [38], the reduced VCAM-1 expression is likely the result of reduced TNF-α expression. In turn, the reduced VCAM-1 and MCP-1 expression in lesions may likely contribute, at least in part, to the reduced numbers of pro-inflammatory macrophages, dendritic cells, CD4 T cells and CD8 T cells in the lesions directly and to the reduced proinflammatory cytokines produced by these cells indirectly. Given that B2 cells have been ascribed antigen-presenting functions, it is likely that the depletion in B2 cells directly contributes to the reduced entry of CD4 and CD8 T cells into the lesions.

In addition to pro-inflammatory cytokines, actively proliferating cells are an indicator for progression of advanced atherosclerosis. In early and progressing atherosclerotic plaques, intimal macrophages are principal proliferative cells constituting about 50% of cell proliferation as assessed by the proliferative cell nuclear antigen antibody [15] whilst three dominant PCNA+ cell types found in advanced plaques are macrophages, vascular endothelial cells and smooth muscle cells. The reduced PCNA+ cells in intimal atherosclerotic lesion in *BaffR.ApoE* DKO mice suggest less proliferative activity in atherosclerotic lesions.

Control of inflammation comes to the forefront in the future management of atherosclerosis. In a meta-analysis of studies on the relationship between methotrexate and cardiovascular disease, methotrexate was associated with a lower risk for CVD patients with chronic inflammatory diseases, suggesting that direct treatment of inflammation will reduce cardiovascular events [39]. Currently the use of BAFF antagonists to target B cells to modulate immune responses in autoimmune diseases is being trialled and shows promising results in animal and human studies [9], [40]–[43].

Given that we have provided compelling evidence that BAFF-R deficiency is associated with potent reduction in inflammation in atherosclerotic lesions, we suggest that targeting the BAFF-R has potential for therapeutic application in the management of patients with atherosclerotic vascular disease.

## Materials and Methods

### Animals

C57Bl/6 mice deficient in both BAFF-R and ApoE genes were generated from crossing BAFF-R^−/−^ mice (from Klaus Rajewsky of the Harvard Medical School) and ApoE^−/−^ mice (from the Jackson Laboratory). Experimental mice (6–8 week- old male mice) were fed a high fat diet containing 21% fat and 0.15% cholesterol (Specialty Feeds, Western Australia) for eight weeks.

### Ethics statement

All animal experiments approved by the Alfred Medical Research and Education Precinct (AMREP) animal ethic committee (E/0708/2008/B) were carried out at the Precinct Animal Centre, AMREP.

### PCR-genotyping of BAFF-R and ApoE genes

Genomic DNAs were extracted from tail samples using DNeasy blood and tissue kit (Qiagen, Germany). PCR application was carried out with 50 ng of genomic DNA in a single reaction vessel containing 20 mM Tris-HCl pH 8.4, 50 mM KCl, 1.5 mM MgCl_2_, 0.2 mM dNTP, 0.2 uM of each primer and .2 unit of Taq DNA polymerase (Invitrogen). PCR condition was as follow: initial denaturation step of 5 minutes at 95°C, 35 cycles of 10 seconds at 95°C, 30 seconds at 60°C and 30 seconds at 72°C and final amplification step of 5 minutes of 72°C. Primers used were ApoE-Com 5′-GCC TAG CCG AGG GAG AGC CG-3′; ApoE-WT 5′- TGT GAC TTG GGA GCT CTG CAG C-3′; ApoE-KO 5′- GCC GCC CCG ACT GCA TCT-3′; BAFF-R-Com 5′-TTC TTT GAG CGG AGG CCA GG-3′; BAFF-R-WT 5′-CTG AGG GAG ACC TGG AGT TG-3′ and BAFF-R-KO 5′-ATG GGC CAC TCA AGA TGA TCT G-3′ (Genework, Australia). PCR products were visualized on ethidium-stained 1.5% TAE agarose gel electrophoresis and digitally recorded.

### Lymphocyte analysis

Lymphocytes were analyzed using flow cytometry as described [5]. Flurochrome-labeled antibodies (all from BD Biosciences unless otherwise stated) used were anti-CD22 (PE-Cat# 553384), anti-CD5 (APC-Cat# 550035), anti-CD25 (APC-Cy7-Cat# 557658), anti-CD4 (Pacific Blue-Cat# MCD0428, Caltag Laboratories), anti-CD8 (PerCP-Cat# 553036), anti-TCR-β (FITC-Cat# 11-596185, eBioscience), anti-NK1.1 (PE-Cy7-Cat# 552878) and anti-foxp3 (PE-Cat# 12-5773-82, eBioscicence). For surface markers, single cell suspension was stained with multiple antibodies at 4°C for 30 minutes, washed away unbounded antibodies and suspended in PBS with 1% FCS. For regulatory T cells, anti-CD4 and anti-CD25 stained cells were fixed, permeabilized and further stained with anti-foxp3 antibody. FACS CantoII (BD Biosciences) was used to collect data from different flurochrome-labeled cells. FACSDiva software (BD Biosciences) was used to analyze the data.

### Assessment of atherosclerosis

To assess the atherosclerosis, the aortic roots embedded in OCT media were sectioned at 6 µ thickness and stained with Oil Red O (Sigma) to visualize lipid accumulation in aortic intima as previously described by us [5]. Immunohistological staining was also carried with anti-CD68 antibody (Cat# MAC1957, Serotec) to assess macrophage accumulation in the atherosclerotic lesions [5]. The lesion assessments were carried out by quantifying the Oil Red O stained lipid accumulation and CD68+ macrophage accumulation by Optimas software [5].

### Lesion cellular content analysis

Immunohistochemical staining was performed on aortic root sections. Anti-mouse antibodies targeting CD4 (Cat# 550280, BD Pharmigen), CD8 (Cat# 550281 BD Pharmigen), CD22 (Cat# 553382, BD Pharmigen), CD83 (Cat# 14-0831, eBioscience), CD11c (Cat# 14-0114, eBioscience), VCAM-1 (Cat# 550547, BD Pharmigen), PCNA (Cat# ab2426, Abcam), IgM (Cat# 550588, BD Pharmigen), IgG1 (Cat# 559626, BD Pharmigen) and IgG2a (553391, BD Pharmigen) were used in primary antibody incubation, followed by horse radish peroxidase-conjugated secondary antibody for DAB substrate. All slides were counter stained with H&E stain. Areas staining by CD11c, VCAM, IgM, IgG1 and IgG2a antibodies were quantified by Optimas software whilst cells stained with CD22, CD4 and CD8, CD83 and PCNA were counted under light microscopy [5], [44]. Both positive areas and positive cells were corrected to total lesion areas as quantified by Optima software.

### Plasma lipid analysis

Determination of plasma cholesterol and triglycerides was done using Beckman Coulter LX20PRO analyzer according to manufacturer's instructions.

### Immunoglobulin analysis

Plasma immunoglobulin IgG1 and IgG2a were measured by enzyme linked immnosorbent assay (ELISA). Nunc Maxisorp 96-well ELISA plates were coated with 50 µl of 2 mg/ml goat anti-mouse Ig antibody (Cat# 1010-01, Southern Biotech) overnight at 4°C. After addition of 50 µl of plasma diluted at 10^5^, the plates were incubated for 1 hour at room temperature. Secondary antibody incubation was done for 1 hour at room temperature with HRP-conjugated goat anti-mouse IgG1 (Cat# 1070-05, Southern Biotech) and IgG2a (Cat# 1080-05, Southern Biotech) antibodies for respective antibody measurement. TMB substrate was used for color development. After stopping the reaction, optic density was measured at 450 nm wave length using ELISA reader. Plasma IgM was measured using rabbit polyclonal anti-mouse Ig antibody (Cat Z0259, Dako) as coating antigen and HRP-conjugated goat anti-mouse IgM (Cat GM-90P, ICL) as detection antibody as previously described [7].

### Real-time analysis of different mRNA

Aortas containing aortic arch and thoracic aorta were immediately frozen in liquid nitrogen. According to manufacturer's instructions, total RNA were extracted from aortas using RNeasy fibrous tissue mini kit (Qiagen, Germany). RNA integrity and quantity were determined using MultiNA electrophoresis system (Shimadzu, Japan). One-step real-time PCR was carried out with QuantiFast SYBR Green RT-PCR kit (Qiagen, Germany) on 7500 Fast Real-Time PCR system (Applied Biosystem). The target gene expression levels were analyzed using comparative cycle threshold method [45] with 18S rRNA primers (Applied Biosystems). The primers used were as follows: IFN-γ sense (S) 5′-AAG TTT GAG GTC AAC AAC CCA C-3′, IFN-γ antisense (AS) 5′-GCT GGC AGA ATT ATT CTT ATT GGG-3′; TNF-α (S) 5′- TCT CAG CCT CTT CTC ATT CCT-3′, TNF-α (AS) 5′- ACT TGG TGG TTT GCT ACG AC-3′; MCP-1 (S) 5′-CTC AGC CAG ATG CAG TTA ACG-3′, MCP-1 (AS) 5′-GGG TCA ACT TCA CAT TCA AAG G-3′; IL1-β (S) 5′-CCA CCT CAA TGG ACA GAA TCT CAA-3′, IL1-β (S) 5′-GTC GTT GCT TGG TTC TCC TTG T-3′.

### Statistical analysis

SigmaPlot 10.0 was used for statistical analyses. Results are presented as mean ± SEM. Two-tailed unpaired student t tests with Welch's correction were used for statistical analyses. *P* values were considered significant at *P*<0.05.
